# Lower-Limb Joint Coordination Pattern in Obese Subjects

**DOI:** 10.1155/2013/142323

**Published:** 2012-12-19

**Authors:** Alberto Ranavolo, Lorenzo M. Donini, Silvia Mari, Mariano Serrao, Alessio Silvetti, Sergio Iavicoli, Edda Cava, Rosa Asprino, Alessandro Pinto, Francesco Draicchio

**Affiliations:** ^1^Department of Occupational Medicine, INAIL, Via Fontana Candida 1, Monte Porzio Catone, 00040 Rome, Italy; ^2^Department of Experimental Medicine, Medical Physiopathology, Food Science and Endocrinology Section, Food Science and Human Nutrition Research Unit, Sapienza University of Rome, Ple Aldo Moro 5, 00185 Rome, Italy; ^3^Villa delle Querce Clinical Rehabilitation Institute, Unit of Metabolic and Nutritional Rehabilitation, Via delle Vigne 19, Nemi, 00040 Rome, Italy; ^4^Fondazione Don Gnocchi, 20148 Milan, Italy; ^5^Rehabilitation Centre, Policlinico Italia, Piazza del Campidano 6, 00162 Rome, Italy; ^6^Department of Medical and Surgical Science and Biotechnologies, Sapienza University of Rome, Via Faggiana 34, 40100 Latina, Italy

## Abstract

The coordinative pattern is an important feature of locomotion that has been studied in a number of pathologies. It has been observed that adaptive changes in coordination patterns are due to both external and internal constraints. Obesity is characterized by the presence of excess mass at pelvis and lower-limb areas, causing mechanical constraints that central nervous system could manage modifying the physiological interjoint coupling relationships. Since an altered coordination pattern may induce joint diseases and falls risk, the aim of this study was to analyze whether and how coordination during walking is affected by obesity. We evaluated interjoint coordination during walking in 25 obese subjects as well as in a control group. The time-distance parameters and joint kinematics were also measured. When compared with the control group, obese people displayed a substantial similarity in joint kinematic parameters and some differences in the time-distance and in the coupling parameters. Obese subjects revealed higher values in stride-to-stride intrasubjects variability in interjoint coupling parameters, whereas the coordinative mean pattern was unaltered. The increased variability in the coupling parameters is associated with an increased risk of falls and thus should be taken into account when designing treatments aimed at restoring a normal locomotion pattern.

## 1. Introduction 

Obesity is a pathology with multifactorial causes that is characterized by an increase in fat body mass and is linked to a significant increase in morbidity and mortality. It is related to the interaction of erroneous eating habits, reduced energy consumption, and metabolic alterations [1]. In obese subjects, body movements are affected by the excess mass, which alters the individual's range of motions and exerts excessive joint load, thereby causing a high incidence of musculoskeletal disorders [2]. Functional tests demonstrated that obese subjects have difficulties in performing activities of daily life and experience more pain than normal-weight individuals [3].

As locomotion is one of the most important and frequent tasks in daily life, gait has been extensively analyzed in previous studies; some of which demonstrated that obesity alters the body's motor scheme, in terms of time-distance, kinematic, and kinetic parameters [4, 5]. Obese adults walk with a wider support base and a lower speed, cadence, and stride length than normal people [6, 7]. Differences emerged between obese and nonobese subjects walking at a standard gait speed in the angular kinematics of the lower-limb joints [8], and in particular reductions in the hip, knee, and ankle range of motions (ROM) on the sagittal plane [6]. By contrast, lower-limb joint kinematic parameters in obese subjects walking at a self-selected velocity were found to be similar to those of healthy subjects [9].

Kinetic analyses revealed that ankle torque is higher in obese than in nonobese individuals walking at a standard speed, whereas, at a self-selected speed, obese individuals produce a gait pattern with lower knee torque and power [8]. Moreover, Malatesta et al. [10] hypothesized that obese people adopt a slower walking speed to reduce the mechanical effort exerted upon the lower extremity muscles and thus minimize energy cost during walking. 

The energetic cost of walking has also been studied in relation to adding mass to the legs; it has been demonstrated that net metabolic rate during walking increases with load magnitude and more distal leg-load location, while there is a small increase in net metabolic rate with proximal loading [11].

A common difficulty of all these studies is the proper placement of passive markers in obese subjects, due to the excessive adipose tissue in the abdominal and pelvic areas. Despite efforts including manual measure of anterior superior iliac spines width [9, 12], potential errors in calculation of hip joint center are possible, as reported in a previous study [13]. Furthermore, another possible source of errors has been reduced by the use of elastic band around the waist to minimize the oscillations of adipose tissues during walking [5, 14].

Although gait pattern alterations in obese people are widely known, no information is, to our knowledge, yet available on the relationship between obesity and lower-limb joint coordination during walking. 

Joint coordination is usually investigated by means of the Continuous Relative Phase (CRP) according to the dynamic system theory [15]. The CRP provides a measure of coupling or phase relationship between the actions of couples of interacting joints or segments and is frequently used to investigate lower-limb coordination in both walking and running [16–19]. 

Since multijoint coordination impairment accompanies numerous pathologies, the CRP has also been used to analyze the effects on gait coordination of numerous musculoskeletal [20, 21] and neurological diseases [22–25]. 

In this study, we hypothesised that the excess mass in the pelvic and lower-limb areas in obese subjects may represent a mechanical constraint that the central nervous system (CNS) is forced to control by means of a coordinative compensatory strategy. This strategy may be aimed at reducing the number of degrees of freedom that the CNS has to control by adopting a more in-phase coupling relationship between pairs of joints.

Since a modified coordination pattern may be a condition predisposing to joint injuries, diseases altering motor control and causing risk of falls [26–28], the aim of this study was thus to evaluate how obesity affects coordination during locomotion using the CRP method. We also analysed the time-distance parameters and lower-limb joint kinematics in the same subjects.

## 2. Materials and Methods

### 2.1. Description of Obese Subjects and Controls

Twenty-five obese subjects (BMI range: 33.8–44.0 kg/m^2^) were enrolled in the study. Twenty-five controls (BMI range: 19.0–27.8 kg/m^2^), gender- and age-matched volunteers, were recruited as a control group (see Table 1). 

None of the controls volunteers had pathologies known to influence the normal gait pattern. Exclusion criteria were severe cardiovascular disease, neurological impairment and lower extremity trauma, lower extremity surgery, and appreciable leg discrepancy. All the participants gave their written consent. The study was approved by the local ethics committee and conformed to the Helsinki declarations. No information regarding the expected results was provided in order to avoid the results being biased, whether consciously or unconsciously.

Both controls and obese subjects underwent anthropometric and functional examinations (Table 1). Anthropometric measurements were performed and body composition was assessed. The anthropometric measurements were based on body weight and stature (SECA scale, Hamburg, Germany) for the calculation of the BMI and waist circumference (measured with an inextensible tape measure midway between the lower rib margin and the iliac crest). As regards body composition, the Siri equation was applied to estimate percentage of fat mass [29]. In addition to the afore-mentioned measurements, a “6-minute walking test” (6MWT) [30], a Borg's Perceived Exertion Scale [31], muscular strength of the forearm flexor muscles [32], and the standing trunk flexibility evaluations were performed in order to assess the functional condition. Muscular strength, expressed in kg, was evaluated by means of the Lafayette dynamometer. Trunk flexibility, defined as the distance between the fingertips and the floor, was evaluated by asking the subjects to reach down towards the floor in front of their feet as far as possible while standing with knees in an extended position. 

### 2.2. Instrumental Evaluation

An optoelectronic motion analysis system (SMART-E System, BTS, Italy), consisting of eight infrared cameras (operating at 120 Hz), was used to detect the movements of spherical markers (15 mm diameter) covered with aluminium powder reflecting material placed over prominent bony landmarks on the skin according to Davis's protocol [33]. Meticulous attention was paid to marker placement in obese patients. If necessary, an elastic band was placed around the waist to avoid any possible movement of adipose tissue that could alter the marker trajectories [14]. All the subjects were asked to wear only underwear or shorts and a tight fitting undershirt. The use of minimal clothing was designed to ensure the correct placement of the markers over the anatomical landmarks. The calibrated walking volume consisted of a level surface that was approximately 6 m in length, with a width of 1.60 m and a height of about 2.00 m. Experiments began with a standing trial, in which rest joint angular displacements were acquired. Obese subjects and controls were then instructed to walk barefoot at a self-selected speed along the level surface. Assuming that this speed would be slower in the obese subjects, we instructed the controls to walk at low speed, too; in this way, gait characteristics could be compared between the groups without the potential velocity bias. Before formal measurements started, subjects did a practice session to familiarize themselves with the experimental procedure by walking for one hour (with some pauses to avoid fatigue). In the experimental session, which was performed the following day, twelve valid trials were acquired for each subject. A valid trial was defined as one in which marker trajectories were not lost during the subject's gait and included at least one cycle per limb. The 1st and 12th trials of each subject were discarded to reduce movement variability related to the start and end of the session. A one-minute rest period was given between groups of 3 trials to avoid fatigue. 

### 2.3. Data Analysis

In the present study, a set of time-distance, kinematic, and coordination parameters were adopted to provide a thorough analysis of gait. Since walking asymmetries were not analyzed in this work, right and left leg data were considered together, yielding a set of generic lower-limb parameters.

#### 2.3.1. Time-Distance Data

A stride was considered as the time between two consecutive heel-floor contacts of the same limb and was subdivided in a stance phase (from 1st initial contact to foot-off) and a swing phase (from foot-off to 2nd heel contact). The double support phase within the stride, defined as the time spent by subjects with both feet on the ground, was also considered. A step was defined as the time between a heel-floor contact of one limb and the consecutive heel-floor contact of the other limb. As time-distance gait parameters, we evaluated stride duration, mean speed, cadence, duration of the stance, swing and double support phases within the stride (all evaluated as percentages of the stride duration), step length, and step width.

For each subject, the time-distance parameters were obtained by averaging the data of the valid strides of 10 successful trials. 

#### 2.3.2. Kinematic Data

Kinematic data were derived using the BTS Smart Analyzer software. Data were smoothed using a triangular four-order window filter. Joint centres of rotation were determined and joint excursions were calculated. Joint angular displacement data were normalized to the stride duration and reduced to 100 samples using a polynomial procedure, thereby defining a gait cycle. We evaluated pelvic tilt, obliquity and rotation, hip flexion-extension, abduction-adduction and rotation, knee flexion-extension, ankle dorsiplantar flexion, and foot progression. For each angle, we calculated the range of motion (ROM), defined as the differences between the maximum and minimum values during the gait cycles. 

#### 2.3.3. Coordination Parameters

Inter-joint coordination on the sagittal plane was assessed by using the CRP technique [21, 34, 35]. A custom written Matlab code (version 7.0; The Mathwoks Inc., MA) was used to compute the coordination parameters. First, the so-called phase portrait of each sagittal joint motion was generated by plotting its normalized angular velocity (*ω*_*N*_) against its normalized angular position (*θ*_*N*_) [35]. The following equations were used to normalize each gait cycle:

(1) $$\begin{array}{c} \omega_{iN}=\frac{\omega_{i}}{\max\left[\max\left(\omega_{i}\right),\max\left(-\omega_{i}\right)\right]},\\ \theta_{iN}=\frac{2*\left[\theta_{i}-\min\left(\theta_{i}\right)\right]}{\max\left(\theta_{i}\right)-\min\left(\theta_{i}\right)}-1, \end{array}$$

where *i* is the percentage of the entire movement cycle, while *θ* and *ω* are, respectively, the angular displacement and angular velocity.

Once the phase portrait had been obtained, the phase angle (*φ*_*i*_) of the joint motion was computed as follows:

(2) $$\begin{array}{c} \varphi_{i}=\tan^{-1}\frac{\omega_{iN}}{\theta_{iN}}, \end{array}$$

where *i* is the time point within the cycle. The CRP angle (*ϕ*_*i*_) between each pair of sagittal joint motions was computed by subtracting the value of the phase angle of the distal joint (*D*_*J*_) from the value of the phase angle of the proximal joint (*P*_*J*_):

(3) $$\begin{array}{c} \phi_{i}=\varphi_{iP_{J}}-\varphi_{iD_{J}}. \end{array}$$

The CRP angles can range between −360° and 360°, with 0° ± 360° indicating in-phase coupling, and −180° and 180° indicating out-phase coupling. A positive CRP indicates that the proximal joint leads the distal. According to the method of Stergiou et al. [36], in order to analyze significant differences between CRP curves, two indices were used for each pair of joint relative movements. The first index is the mean absolute relative phase (MARP), which was computed by averaging the absolute values of the ensemble curve points for both the stance and the swing phases:

(4) $$\begin{array}{c} \text{MARP}=\frac{\sum_{i=1}^{p}\left|{\overset{-}{\phi }}_{i}\right|}{p}, \end{array}$$

where *p* is the number of time points in each phase. The second parameter is the deviation phase (DP), which is calculated by averaging the standard deviations of the ensemble CRP curve points for both the stance and the swing phases:

(5) $$\begin{array}{c} \text{DP}=\frac{\sum_{i=1}^{p}SD_{i}}{p}. \end{array}$$

DP provides a measure of stability of the organization of the neuromuscular system. A low DP value indicates a less variable intrasubjects stride-to-stride relationship between the actions of the two joints. Three couplings of joints were considered: hip-knee, knee-ankle, and hip-ankle. The MARP_stance_, DP_stance_, MARP_swing_, and DP_swing_ on the sagittal plane were calculated for each of these couplings of joints.

#### 2.3.4. Statistical Analysis

The statistical analysis was performed using PASW software (PASW Statistic 17, Chicago, USA). Means and standard deviations were calculated for time-distance, kinematic, and coordination parameters. For each variable, the Shapiro-Wilk test was performed to assess the Gaussian distribution of the two samples. Then, the 2-tailed *t*-test for equality of means was applied when the parameters were found to be normally distributed. The nonparametric test (Mann-Whitney) was performed for non-Gaussian variables. We considered *P* values of less than 0.05 as statistically significant. 

To evaluate the group effect on the mean joint coupling variables we calculated the between-group coefficient of multiple correlation (CMC_BG_) between the mean waveforms of the obese and the control groups. To determine the level of homogeneity within each group, we also calculated the within-group coefficient of multiple correlation (CMC_WG_) between the mean waveforms of the subjects in the obese group and in the healthy group. 

CMC, that is, the positive square root of the adjusted coefficient of multiple determination [37, 38], is a measure of the overall waveform similarity of a group of curves; the closer to 1 the CMC is, the more similar the waveforms are. We calculated the two CMC as follows:

(6) $$\begin{array}{c} \text{CMC}=\sqrt{1-\frac{\left(1/\left(T\left(N-1\right)\right)\right)\sum_{i=1}^{N}\sum_{t=1}^{T}{\left(y_{it}-{\overset{-}{y}}_{t}\right)}^{2}}{\left(1/\left(TN-1\right)\right)\sum_{i=1}^{N}\sum_{t=1}^{T}{\left(y_{it}-\overset{-}{y}\right)}^{2}}}, \end{array}$$

where *T* = 100 (number of time points within the cycle), *N* is the number of curves (2 for CMC_BG_ and 25 for CMC_WG_), *y*_*it*_ is the value at the *t*th time point in the *i*th curve, ${\overset{-}{y}}_{t}$ is the average at time point *t* over *N* curves:

(7) $$\begin{array}{c} {\overset{-}{y}}_{t}=\frac{1}{N}\sum_{i=1}^{N}y_{it} \end{array}$$

and $\overset{-}{y}$ is the grand mean of all *y*_*it*_:

(8) $$\begin{array}{c} \overset{-}{y}=\frac{1}{NT}\sum_{i=1}^{N}\sum_{t=1}^{T}y_{it}. \end{array}$$

## 3. Results 

As correctly assumed, obese subjects showed a significantly reduced mean gait speed compared to controls when walking at self-selected speed (0.93 ± 0.11 m/s versus 1.138 ± 0.14 m/s, *P* = 0.001). However, obese subject mean self-selected speed was not statistically different from that of controls when walking at low speed (0.93 ± 0.11 m/s versus 0.92 ± 0.19 m/s, *P* = 0.794). Thus, in order to avoid the potential velocity bias, time-distance, kinematic, and coordination parameters were compared between obese subjects walking at self-selected speed and controls walking at low speed. 

### 3.1. Time-Distance Data

The means and standard deviations of the time-distance parameters and *P*-values are shown in Table 2. Obese subjects spent a significantly greater percentage of the gait cycle in the stance (5% more) and double support (23% more) phases than controls, while their swing phase was shorter (a 7% reduction). Furthermore, the step width was greater (an 30% increase) in the obese group than in controls. 

### 3.2. Kinematic Data

**Figure 1:**
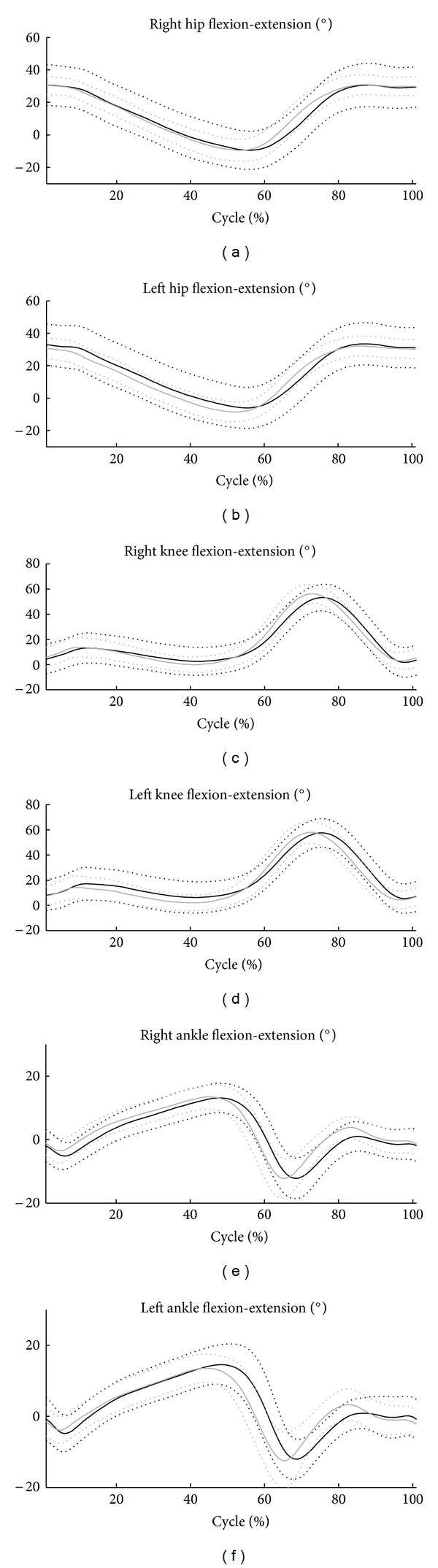
Mean and standard deviation of angular displacement of hip, knee, and ankle joint in the sagittal plane. The black curve refers to obese subjects, the gray one to controls.

The means and the standard deviations of hip, knee, and ankle joint angular ROM on the sagittal plane are shown in Figure 1.  Table 3 shows the means and standard deviations of the kinematic parameters. The table also presents the statistical analysis results. Significant reductions in knee flexion-extension (7%) and pelvic obliquity (24%) ROMs were observed in obese subjects if compared with controls. Lastly, a significant increase in pelvic tilt ROM (17%) was found in obese subjects if compared with controls.

### 3.3. Coordination Parameters

**Figure 2:**
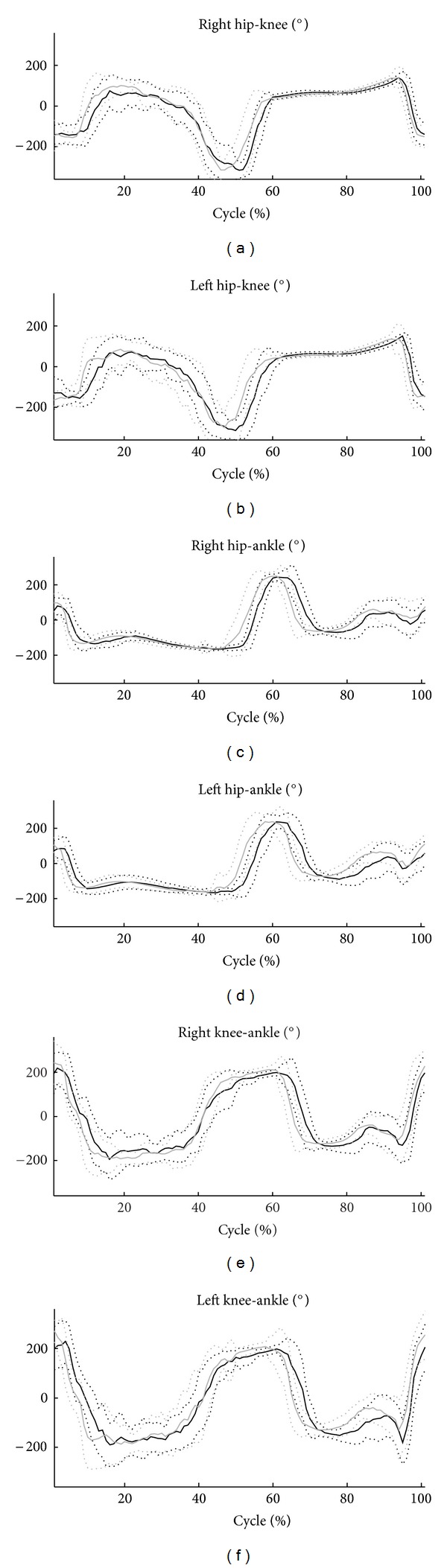
Mean and standard deviation of CRP at the hip-knee, hip-ankle, and knee-ankle joint coupling. The black curve refers to obese subjects, the gray one to controls.

The means and the standard deviations of the ensemble CRP curves are shown in Figure 2. The means and standard deviations of the coordination parameters and the statistical analysis results are reported in Table 4. No significant differences in MARP mean values were observed between the two groups in both stance and swing phases.

By contrast, the joint coordination variability, as calculated by means of the DP, was always greater in obese subjects than in controls. Indeed, an increase of DP values at the hip-knee (stance 40%, swing 17%), hip-ankle (stance 35%, swing 28%), and knee-ankle (stance 40%, swing 19%) couplings were observed.

CMC_WG_ and CMC_BG_ values are summarized in Table 5.

## 4. Discussion

The aim of this work was to evaluate how gait coordination is influenced by obesity. To achieve this aim, we calculated time-distance data, kinematic, and inter-joint Continuous Relative Phase parameters. We observed a substantial similarity between obese subjects and controls in segmental parameters (joint ROMs) and some significant differences between the two groups in the global (time-distance) and inter-joint coupling (CRP) parameters. 

As regards the time-distance parameters, in keeping with the results of previous works [5–7, 38], our findings reveal that the gait cycle in obese subjects is characterized by a longer stance and double support phase, a shorter swing phase, and a wider base of support. This altered gait pattern is probably related to an increased need for stabilization caused by obesity. Indeed, the greater double support and stance phase is likely to provide a safer locomotion by maintaining the weight on both limbs and not overloading one limb, thereby reducing the risk of instability [9]. The increased effort made by obese individuals to maintain body balance also yields high values in step width (due, in part, also to the increased body mass encumbrance between the legs) and in a lower gait self-selected speed. Moreover, the lower gait self-selected velocity may be read as being indicative of poor physical condition in obese adults as well as an attempt to give the CNS more time to react to obstacles [39]. These altered global gait parameters are similar to the ones exhibited by older adults who tend to fall. Indeed, according to Maki [39], changes in the time-distance parameters in obese subjects may also be associated with a preexisting fear of falling. 

From a kinematic point of view, obese subjects displayed a substantial similarity in the angle curves of the pelvis and lower-limb joints within the gait cycle (see Figure 1). However, a significant decrease emerged in obese subjects in pelvic obliquity and in knee joint flexion-extension ROMs, whereas a significant increase was observed in pelvic tilt ROM. The reduced ROMs may be due to the fact that obese subjects need to keep both limbs in contact with the ground to gain stability, which increases the amount of time spent in a closed lower-limb kinematic chain condition. This reduces the degrees of freedom of the rigid lower body system and consequently exerts greater constraint on the pelvic segment and knee joint. Another possible explanation for these reduced ROMs may be the excess weight of the limbs, which represents an extra load for the muscles involved in pelvic obliquity and knee flexion movement during the preswing subphase and swing phase. 

In particular, the reduction in knee ROMs may be due to the presence of excess fat on the thigh and shank, which mechanically encumbers intersegmental rotation [40] and counteracts the antigravity action exerted by the knee flexors. According to Park et al. [40], the main cause of the reduction observed in ROMs in obese subjects is the lower level of daily physical activity, which limits both weight loss and muscle strengthening. These kinematic results are also partially in keeping with those obtained by Spyropolous et al. [6], who reported lower ROMs in obese subjects than in controls walking at a self-selected speed. 

We investigated the coordinative behaviour by means of the CRP technique. The choice of such method instead of others based on principal component analysis [41] was determined by the need to quantify, sample by sample, the coupling relationship between two joints. One of the main findings of our study is that the joint coordination pattern in obese subjects was substantial similar from that observed in nonobese subjects, in both the stance and swing phases. These results indicate that the hip, knee and ankle joints play the same role in obese subjects as in controls in producing a coordinated walking pattern within the gait cycle. The fact that the mechanical constraint exerted by the lower-limb excess mass does not affect the coordinative strategy adopted by the CNS is, however, in contrast to our hypothesis.

Previous studies have shown that in obesity there is a neuromuscular adaptation, implemented by a decreasing self-selected gait speed, which results in reduced ground reaction forces, lower joint loads, and net muscle moments during gait and is aimed at reducing the risk of musculoskeletal diseases [8, 42]. We hypothesize that the afore-mentioned neuromuscular adaptations may be the result of walking control mechanisms designed to preserve a physiological inter-joint lower-limb coupling pattern. This motor behaviour, anchored to the physiological coordinative strategy, results in the high CMC_WG_ and CMC_BG_ values that point to a strong similarity among obese subjects as well as between obese and control subjects in inter-joint lower-limb couplings. 

A surprising finding of the present study is the wide stride-to-stride intrasubject variability (see DP values) in all the joint couplings in obese subjects if compared with controls, which may be a risk factor for falls [43]. As recently showed, gait variability gives indirect information on the control mechanism of locomotion [44]. In particular, a low variability is related to the stability of the locomotion, meaning the capacity to maintain the dynamic balance. 

The higher variability we found may be due to the excess fat distributed above the pelvis and the lower-limb segments, creating a mechanical encumbrance, that has to be continuously managed stride by stride. Since the distribution of this excess fat is not the same in men and women [45], a study designed to analyze gender differences in coordinative strategies may help confirm these results. 

In a recent study by Yen et al. [35], an increased variability in the coupling relationship was observed in elderly people during obstacle crossing. It was read as an age-related biomechanical change associated with a lower ability to maintain a stable body balance, which might increase the risk of falls during walking. For this reason, further studies are warranted to shed light on the relationship in obese people between the risk of falling and inter-joint coordination as well as on the relative position between the centre of pressure and the “extrapolated centre of mass” [46]. 

The CRP curves show that the pattern in obese subjects is topologically similar to that of controls (see Figure 2 and CMC_BG_ in Table 5). The only difference that emerged is a time shift between the mean curves of obese subjects and those of the controls, which is particularly pronounced at the end of the stance phase and during the swing phase of gait. The time shift that is evident in the CRP curves of obese subjects is in keeping with the changes found in the time-distance parameters concerning the increase in the stance phase and the decrease in the swing phase. 

The accuracy of the marker placement in obese subjects is one possible limitation of this work. Indeed, although we adopted strategies to minimize the likelihood of errors, marker trajectory anomalies, especially in pelvis markers, which are subject to movement-induced oscillations due to the excess fat at the waist, cannot be ruled out. For this reason, in the kinematic results, the small differences observed in pelvic ROMs between obese subjects and controls could be considered as not relevant, especially taking into account that the pelvic angular excursions are very small.

In view of the above, the reduction of the variability associated to the lower-limb joint coupling relationships analyzed in the present study may prove useful as indexes to design and assess the effectiveness of dietary treatment, of physical exercise and of rehabilitative protocols adopted.

## 5. Conclusions 

In this study we performed an exhaustive gait analysis to investigate the time-distance, kinematic, and coordinative alterations that occur in obese subjects. The results of this work shed light on the motor strategy adopted by obese individuals, which is aimed at maintaining body balance and at preserving a physiological inter-joint lower-limb coupling pattern. Since obesity was found to be related to an increased intrasubject stride-to-stride coordination variability, the study of joint coordination and specific rehabilitation interventions in walking performance may help to improve activities of daily life in these subjects.

## Figures and Tables

**Table 1:** Means, ranges, standard deviations, and *t*-test significance (*P* values) of the personal, anthropometric, and functional characteristics of the two groups. *P* values lower than 0.05 are shown in bold.

Characteristics	Obese subjects	Controls	*P* values
Sex (*n*)			
M	8	8	
F	17	17	
Age range (years)	34–58	33–59	0.85
BMI range (kg/m^2^)	33.8–44.0	19.0–27.8	**<0.001**
Trunk flexibility (cm)	14.3 ± 13	5.0 ± 6.1	**0.006**
Waist circumference (cm)			
M	133.5 ± 14	95.8 ± 8	**<0.001**
F	113 ± 11	78.2 ± 7	**<0.001**
Fat mass (%)			
M	37.3 ± 6	25.1 ± 3	**0.002**
F	43.1 ± 3	32.2 ± 4	**<0.001**
6MWT (m/s)			
M	1.39 ± 0.2	1.38 ± 0.1	0.85
F	0.97 ± 0.5	1.26 ± 0.4	**0.03**
Borg			
M	3.6 ± 1	2.5 ± 0.6	0.16
F	4.1 ± 1	1.9 ± 1	**<0.001**
Strength (kg)			
M	41.6 ± 14	51.5 ± 6	0.08
F	25.1 ± 8	22.4 ± 9	0.3

**Table 2:** Means, standard deviations of the time-distance parameters, and their statistical significance (*P* values). In-bold *P* values are lower than 0.05.

Time-distance parameters	Obese subjects	Controls	*P* values
Stride duration (s)	1.20 ± 0.11	1.26 ± 0.15	0.183
Mean speed (m/s)	0.93 ± 0.11	0.92 ± 0.19	0.794
Cadence (step/min)	101.70 ± 8.94	96.86 ± 11.09	0.137
Stance %	64.06 ± 1.76	61.22 ± 2.18	**<0.001**
Swing %	35.94 ± 1.76	38.78 ± 2.18	**<0.001**
Double support %	13.77 ± 1.74	11.23 ± 2.02	**<0.001**
Step length (m)	0.49 ± 0.04	0.51 ± 0.05	0.199
Step width (m)	0.26 ± 0.04	0.20 ± 0.06	**<0.001**

**Table 3:** Means, standard deviations, and significance (*P* values) of the kinematic parameters. *P* values lower than 0.05 are shown in bold.

ROM (°)	Obese subjects	Controls	*P* values
Ankle flexion-extension	28.52 ± 5.20	28.35 ± 6.21	0.926
Foot progression	12.57 ± 2.55	13.95 ± 3.49	0.163
Knee flexion-extension	55.09 ± 4.12	58.98 ± 5.48	**0.015**
Hip flexion-extension	42.01 ± 4.83	42.31 ± 4.73	0.841
Hip abduction-adduction	14.42 ± 2.90	13.10 ± 3.12	0.174
Hip rotation	14.86 ± 4.06	14.76 ± 3.40	0.820
Pelvic tilt	3.80 ± 0.87	3.24 ± 0.86	**0.021**
Pelvic obliquity	5.32 ± 1.35	6.99 ± 2.68	**0.018**
Pelvic rotation	8.28 ± 2.65	9.78 ± 2.93	0.097

**Table 4:** Means, standard deviations, and significance of the coordination parameters. Significant results are shown in bold. H: hip, K: knee, A: ankle.

Coordination parameters (°)	Obese subjects	Controls	*P* values
Stance MARP H-K	125.33 ± 10.76	123.77 ± 11.06	0.654
Stance DP H-K	73.90 ± 20.08	52.88 ± 22.87	**0.001**
Stance MARP H-A	139.83 ± 9.36	142.89 ± 12.32	0.382
Stance DP H-A	37.35 ± 10.20	27.76 ± 7.30	**0.000**
Stance MARP K-A	155.19 ± 12.93	160.48 ± 14.15	0.180
Stance DP K-A	72.11 ± 19.24	51.39 ± 21.96	**0.001**
Swing MARP H-K	88.53 ± 7.48	91.16 ± 7.90	0.288
Swing DP H-K	22.16 ± 5.66	18.99 ± 7.74	**0.015**
Swing MARP H-A	74.67 ± 25.34	72.49 ± 17.82	0.755
Swing DP H-A	61.57 ± 19.00	48.28 ± 19.45	**0.035**
Swing MARP K-A	120.74 ± 26.70	112.45 ± 18.35	0.260
Swing DP K-A	68.47 ± 15.78	57.46 ± 17.17	**0.041**

**Table 5:** CMC_WG_ values for obese subjects and controls and CMC_BG_ values. H: hip, K: knee, A: ankle.

Couples of joints	CMC_WG_ obese subjects	CMC_WG_ controls	CMC_BG_
H-K	0.885	0.845	0.975
H-A	0.885	0.870	0.940
K-A	0.880	0.875	0.965
